# Genetic insights into hypertrophic cardiomyopathy: pathogenesis, diagnosis, and therapeutic implications

**DOI:** 10.1186/s44348-025-00055-4

**Published:** 2025-07-21

**Authors:** Eui-Young Choi, Hyemoon Chung, Kyung-A. Lee

**Affiliations:** 1 Division of Cardiology, Department of Internal Medicine, Gangnam Severance Hospital, Yonsei University College of Medicine, Seoul, Republic of Korea; 2 Division of Cardiology, Department of Internal Medicine, Kyung Hee University Medical Center, Seoul, Republic of Korea; 3 Department of Laboratory Medicine, Gangnam Severance Hospital, Yonsei University College of Medicine, Seoul, Republic of Korea

**Keywords:** Hypertrophic cardiomyopathy, Genetics, Sarcomeric mutations, Mitochondrial variants, Prognosis, Precision medicine

## Abstract

We conducted a comprehensive literature review of sarcomeric gene studies, registry analyses, and recent cohort investigations, focusing on genetic testing outcomes and clinical prognostication. Sarcomeric mutations account for approximately 60% of familial hypertrophic cardiomyopathy (HCM) cases and exhibit variable penetrance and expressivity. Additionally, mitochondrial DNA variants and nonsarcomeric genetic modifiers contribute to the phenotypic heterogeneity observed in HCM. Genetic testing facilitates diagnosis in atypical cases, guides cascade testing in families, and supports reproductive decision-making. Long-term follow-up data from registries indicate that sarcomere-positive patients are diagnosed approximately 13 years earlier and experience nearly double the 50-year incidence of adverse cardiovascular events compared to sarcomere-negative individuals. In Korean cohorts, the mutation detection rate is reported at 43.5%, with genotype-positive status independently associated with worse outcomes. However, for certain prognostic outcomes—particularly sudden cardiac death—more robust data are needed. Emerging therapies, including myosin inhibitors and gene-editing approaches, show promise in targeting the underlying molecular mechanisms of HCM. Therefore, integrating comprehensive genetic screening—including sarcomeric, mitochondrial, and modifier genes—is essential for precise risk stratification and personalized management of HCM. Future efforts should focus on refining variant interpretation and advancing genotype-guided therapeutic strategies.

**Supplementary Information:**

The online version contains supplementary material available at 10.1186/s44348-025-00055-4.

## Background

Hypertrophic cardiomyopathy (HCM) is a prototypical genetic cardiomyopathy. Understanding its genetic background can enhance our insight into its pathogenesis and potential therapeutic targets. While the clinical presentation is variable—from asymptomatic to sudden cardiac death (SCD)—advances in molecular genetics have revolutionized our understanding of HCM pathogenesis [1, 2]. In this review, we aim to synthesize current evidence on the genetic architecture of HCM, including sarcomeric and nonsarcomeric variants, inheritance patterns, genotype–phenotype correlations, and their implications for diagnosis, prognosis, and treatment.

## Role of genetic testing and variant interpretation

Genetic testing is recommended for index HCM cases and at-risk relatives to enable early detection and surveillance. High-throughput panels and whole-genome sequencing enhance detection rates by identifying structural and deep intronic variants, improving diagnostic yield by up to 12% compared to conventional panels [3]. Interpreting variants of uncertain significance (VUS) remains a challenge; standardized workflows incorporating the American College of Medical Genetics and Genomics (ACMG) guidelines, ClinGen expert curation, and computational tools (e.g., CardioClassifier, CardioVAI) help optimize classification accuracy and clinical utility.

## Genetic architecture of HCM

### Sarcomeric gene mutations

Pathogenic variants in sarcomere protein genes account for 60% to 70% of familial HCM cases. Mutations in *MYBPC3* and *MYH7* constitute approximately 80% of identified sarcomeric defects, with *TNNT2, TNNI3, TPM1, ACTC1, MYL2*, and *MYL3* comprising the remainder. These autosomal dominant mutations exhibit variable penetrance, with *MYH7* carriers often displaying earlier onset and more severe hypertrophy compared to *MYBPC3* carriers. A recent reappraisal by the ClinGen Gene Curation Expert Panel reaffirmed the pathogenic relevance of these core sarcomeric genes and newly included *TNNC1* among the curated gene set [4].

### Nonsarcomeric variants

**Fig. 1:**
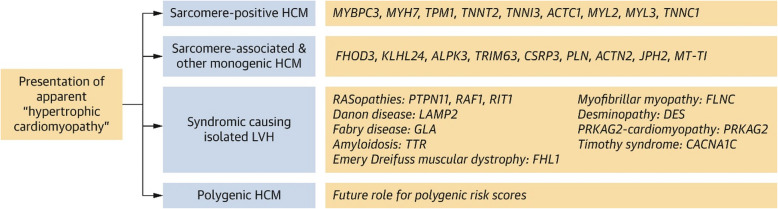
Overview of genetic subtypes of hypertrophic cardiomyopathy (HCM) and associated genes with moderate, strong, or definitive evidence. LVH, left ventricular hypertrophy. Reprinted from Hespe et al. [4], with permission from Elsevier

**Fig. 2:**
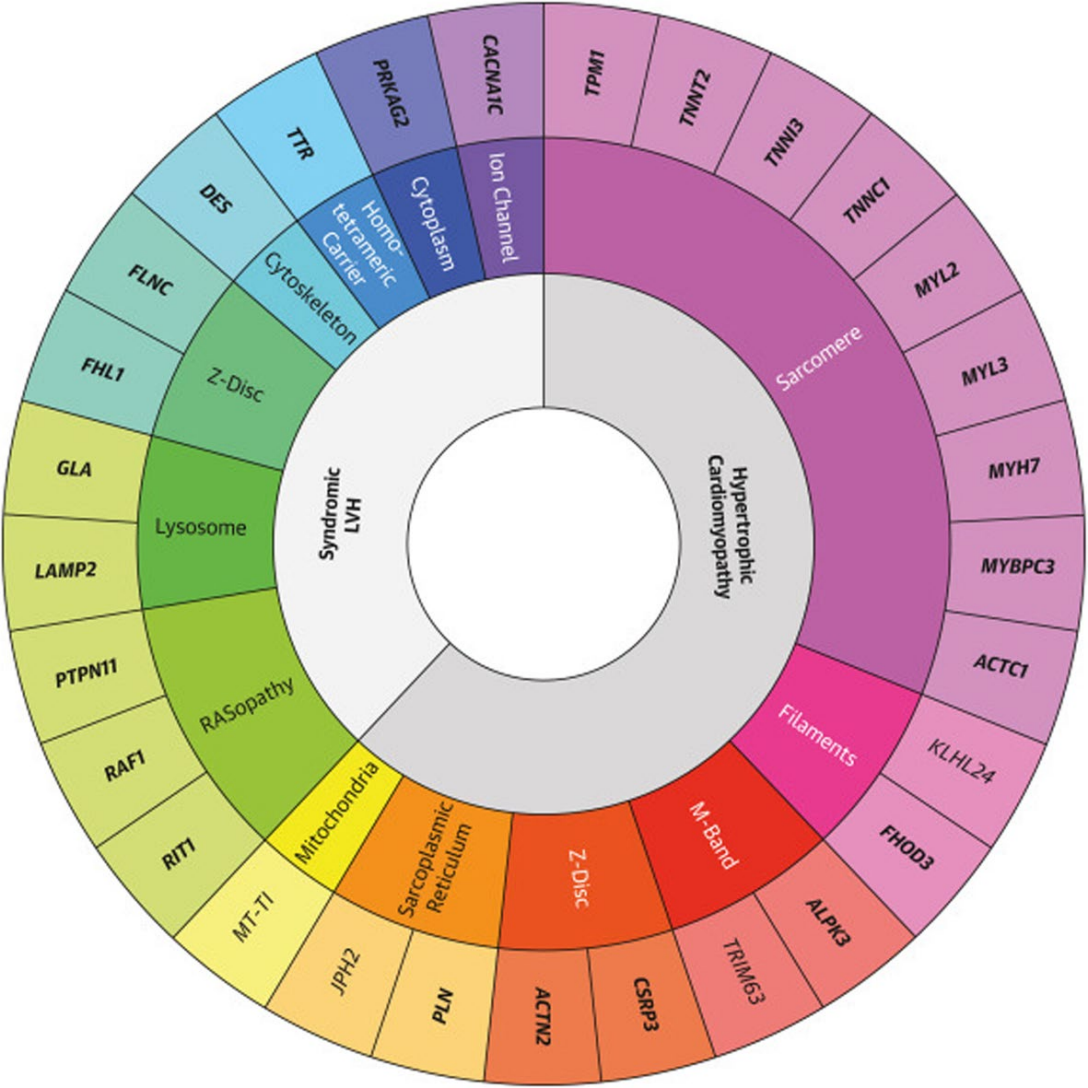
Updated list of genes with moderate, strong, or definitive hypertrophic cardiomyopathy association. LVH, left ventricular hypertrophy. Reprinted from Hespe et al. [4], with permission from Elsevier

Recent US-based studies have further advanced the genetic understanding and therapeutic landscape of HCM. The ClinGen Hereditary Cardiovascular Disease Gene Curation Expert Panel recently reevaluated 31 genes, resulting in the reclassification of 17. Notably, *TNNC1* was upgraded to a definitive HCM gene, while *TRIM63* and *ALPK3* were recognized for their dual inheritance patterns. Additionally, *FHOD3* was newly classified as definitively associated with HCM. This reclassification enhances the precision of genetic testing and variant interpretation in HCM management [4]. ​The panel reported that sarcomere-associated genes with moderate, strong, or definitive evidence include *FHOD3, KLHL24, ALPK3, TRIM63, CSRP3, PLN, ACTN2, JPH2, and MT-TI* [4]. In addition, they identified phenocopy gene variants—such as *GLA, TTR, DES, LAMP2*, and *PRKAG2*—which are associated with syndromic diseases (e.g., Fabry disease or hereditary transthyretin amyloidosis) that can present with myocardial thickening (Figs. 1, 2) [4].

### Mitochondrial genetics

A subset of HCM patients lacks sarcomeric mutations but harbors pathogenic mitochondrial DNA (mtDNA) variants or deep intronic/structural alterations in sarcomeric loci [5]. Privately inherited mtDNA haplogroups and transfer RNA (tRNA) mutations further modulate disease susceptibility [4, 6, 7]. Recent Korean studies have identified mitochondrial variants, including *MT-RNR2*, associated with apical HCM [6]. Emerging evidence highlights the role of mitochondrial dysfunction and mtDNA variants in the pathogenesis of genotype-negative HCM. One study demonstrated that mitochondrial dysfunction correlates with septal hypertrophy in genotype-negative cases, suggesting the potential utility of mitochondria-targeted therapies and advocating for the inclusion of mitochondrial genome sequencing in HCM genetic evaluation [8]. Investigations into mitochondrial respiratory function in HCM have shown that genotype-negative patients exhibit impaired oxidative phosphorylation and specific deficiencies in complexes I and IV, implicating mitochondrial dysfunction as a potential primary driver in these cases [9]. Early reports of HCM attributable to mtDNA mutations described point mutations that led to cardiomyopathy confirmed by pathology and sequencing, establishing the concept that mtDNA variants can directly cause HCM [7]. Population studies have also identified private mtDNA variants as susceptibility factors for HCM, with certain haplogroups showing increased risk—underscoring the importance of considering mtDNA in genetic risk assessments [10]. Furthermore, pathogenic variants in mitochondrial tRNA genes (e.g., *MTTK, MTTL1*) have been associated with hypertrophic phenotypes in mitochondrial cardiomyopathies, reinforcing the need to screen for tRNA mutations in comprehensive HCM evaluations [11].

### Rare and common variant contributions to HCM risk

A cohort study integrating exome sequencing and the Genome-Wide Association Study data from the UK Biobank and the Mass General Brigham Biobank demonstrated that pathogenic rare variants in 14 core ACMG-designated genes conferred up to a 55-fold increased risk of HCM (e.g., *MYH7* odds ratio [OR], 61; *MYBPC3* OR, 72). In parallel, a polygenic risk score derived from 27 common variants independently improved risk prediction (OR per standard deviation, 1.6), jointly increasing the area under the receiver operating characteristic curve from 0.71 to 0.82 when combined with clinical factors [12]. Bagnall et al. [5] demonstrated that incorporating whole-genome sequencing in HCM diagnostic workflows increased variant detection rates by 12% over standard panels, uncovering deep intronic and structural variants in *MYBPC3* and *TNNT2* which were previously missed, and influenced clinical management in 8% of cases. Lopes et al. [13] used high-throughput sequencing of 41 genes in a UK cohort (n = 150) and identified novel genotype–phenotype associations, showing that additional nonsarcomeric variants modulate disease severity in sarcomere mutation carriers, and that compound heterozygosity was linked to earlier onset and more severe hypertrophy. A recent large-scale genome-wide association and multitrait analysis including 5,900 HCM cases and over 68,000 controls identified 70 loci, of which 50 were novel, associated with HCM risk and cardiac magnetic resonance imaging traits. These findings underscore the polygenic architecture of HCM in European populations and support integrating polygenic risk scores alongside monogenic testing for risk stratification [14].

## Clinical utility of genetic test

Genetic testing facilitates diagnosis in atypical cases, guides cascade testing in families, and supports reproductive decision-making. It can help differentiate thickened myocardium, such as in phenocopy diseases (e.g., cardiac amyloidosis, sarcoidosis, Fabry disease or syndromic cardiomyopathies) (Figs. 1 and 2) [4], hypertensive heart disease, and athlete’s heart. Most importantly, it guides family surveillance by cascade testing (Table 1) [1]. For pregnancy planning, during genetic counseling, it should be clearly communicated to the proband and their partner about the inheritance rate and the potential for phenotypic development. Identifying a pathogenic variant allows for preimplantation genetic testing. In this process, embryos are created via in vitro fertilization, and those not carrying the pathogenic variant are selected for implantation. However, due to ethical concerns, this technique is not widely performed [15].

**Table 1:** Genetic testing strategy and surveillance recommendations for hypertrophic cardiomyopathy families

Category of proband	Genetic testing	Surveillance recommendation
Pathogenic/likely pathogenic variant	Offer to all first-degree relatives	ECG and TTE every 1–2 yr for children and 3–5 yr for adults if genotype-positive but phenotype-negative No further surveillance if genotype-negative
VUS	Counsel on uncertainty; consider cascade testing with caution	Surveillance based on phenotype, reclassification suggested
Negative genetic test	No further testing for relatives	Informed to return for evaluation if they experience clinical changes suggestive of cardiac disease
Genetic test not done		ECG and TTE every 1–2 yr for children and 3–5 yr for adults

Based on the 2024 American Heart Association/American College of Cardiology guidelines [1]. If the variant is absent, it should be informed that any changes in variant interpretation may prompt a return to screening

ECG, electrocardiography; TTE, transthoracic echocardiogram; VUS, variants of uncertain significance

## Genotype–phenotype correlations

Certain mutations correlate with distinct phenotypes. *MYH7* mutations tend to produce earlier and more severe hypertrophy. ​*MYBPC3* mutations are associated with late-onset disease and may exhibit incomplete penetrance. ​Mutations in *TNNT2* are often linked to a high risk of SCD despite minimal hypertrophy. ​

### Genetic contributions to HCM phenotypes in Korean data

Chung et al. [6] conducted a comprehensive genetic analysis involving 212 HCM patients, examining 82 nuclear DNA (sarcomere associated genes, phenocopy genes and mitochondria related genes) and 37 mtDNA variants. Their study revealed that pathogenic variants in sarcomere-associated genes were more prevalent in nonapical HCM (41.4%) compared to apical HCM (20.8%). Interestingly, apical HCM exhibited a higher frequency of *TNNI3* variants (35%) and a lower frequency of *MYH7* variants (9%). Additionally, mitochondrial gene *MT-RNR2* was positively associated with apical HCM, suggesting a distinct genetic profile for this subtype. These findings highlight the heterogeneity of HCM and underscore the importance of considering both sarcomere and mitochondrial gene variants in understanding its phenotypic diversity.

### Impact of sarcomere mutations on myocardial fibrosis and mechanics

Several studies confirmed that sarcomere-associated mutations were significantly related to increase in myocardial fibrosis [16, 17]. In addition, Kim et al. [18] investigated the effects of sarcomere mutations on myocardial mechanics and fibrosis patterns using cardiac magnetic resonance imaging and echocardiography. Their study demonstrated that patients with sarcomere mutations had lower left ventricular circumferential strain and higher basal rotation, along with a higher prevalence of mid-wall fibrosis. These alterations in myocardial mechanics and fibrosis patterns were independent of the extent of hypertrophy, indicating a direct impact of genetic mutations on myocardial function. In asymptomatic carriers without left ventricular hypertrophy (LVH) at baseline, those who developed LVH over an 8-year period had reduced peak global strain rate during the isovolumic relaxation period and lower global longitudinal strain (GLS) compared to those who did not develop LVH [19]. In a recent study, myocardial work indices were significantly worse in sarcomere mutation carriers compared to controls, suggesting that myocardial work indices is more sensitive to early changes than GLS and could play an important role in the evaluation and follow-up of carriers [20]. Therefore, left ventricular GLS and myocardial work index may serve as sensitive indicators for detecting early myocardial changes in mutation carriers without overt LVH. This group of family members should be monitored more closely. Regarding diffuse early interstitial fibrosis in carriers without hypertrophy, some studies have shown elevated extracellular volume and abnormal T1 values, suggesting diffuse fibrosis in mutation carriers [21, 22]. These findings support the hypothesis that fibrosis may be a primary phenotype in HCM prior to the development of overt hypertrophy. However, since both studies were conducted by the same research group, further confirmation is needed.

#### Impact of sarcomere mutations on left atrial function

In another study, Chung et al. [23] explored the relationship between sarcomere gene mutations and left atrial function in HCM patients. They found that patients with pathogenic sarcomere mutations exhibited larger left atrial volumes and reduced left atrial emptying fractions, independent of left ventricular filling pressures. This suggests that sarcomere mutations may contribute to atrial myopathy in HCM, potentially influencing the risk of atrial fibrillation and other complications [23, 24]

## Genetic evidence related to clinical course or prognostication

Currently, the evidence supporting the use of genetic testing for prognostication in HCM remains limited. This may be partly due to the uncertainty surrounding disease duration, as many HCM patients remain asymptomatic for extended periods. Additionally, for certain prognostic outcomes—particularly SCD—more robust data are needed. The 2024 American Heart Association/American College of Cardiology guidelines classify the utility of genetic testing for SCD risk stratification as uncertain (class IIb) [1]. Nevertheless, emerging genetic-based studies provide valuable insights that may inform future risk assessment and clinical decision-making.

### Insights from the US and global registry

The Sarcomeric Human Cardiomyopathy Registry analyzed over 4,500 patients and found that sarcomere mutation carriers were diagnosed younger and experienced more adverse outcomes than noncarriers. By age 50 years, 29% of sarcomere mutation carriers experienced major adverse events, compared to 14% of noncarriers. Early diagnosis (before age 40 years) was associated with a significantly greater lifetime burden of disease such as cumulative incidence of major adverse events—comprising heart failure, atrial fibrillation, ventricular arrhythmias, and sudden cardiac death—reached 77% by age 60 years. In contrast, patients diagnosed after age 60 years had a 32% cumulative incidence of such events by age 70 years. Notably, heart failure and atrial fibrillation were the most prevalent complications, often emerging in later decades of life [25]. ​These findings highlight the importance of genetic testing in HCM for risk stratification and management. Identifying pathogenic sarcomere mutations can inform prognosis and guide surveillance strategies. Furthermore, the data suggest that early intervention and continuous monitoring are crucial, especially for patients diagnosed at a younger age, to mitigate the progression to heart failure and atrial fibrillation [25]. Patients with VUS exhibited an intermediate risk profile, suggesting that some VUS may have pathogenic potential [25]​.

### Insights from European studies

Mazzarotto et al. [26] critically reviewed three decades of genetic testing in HCM, highlighting that while pathogenic sarcomeric variants facilitate family screening, ambiguous gene associations and VUS complicate interpretation. They proposed a tiered genetic testing approach focused on a validated panel of sarcomeric genes to enhance diagnostic yield and variant classification accuracy, leveraging standardized ACMG guidelines and resources like ClinGen, CardioClassifier, and CardioVAI. Furthermore, they emphasized that genotype-negative patients often represent non-Mendelian HCM with a comparatively benign prognosis, underscoring the importance of differentiated clinical counseling and management strategies. Although several studies showed that patients with *MYH7* variants had worse outcomes regarding development of atrial fibrillation and progression to advanced heart failure compared to *MYBPC3* mutation carriers [25, 27], an Austrian registry found that patients with *MYBPC3* mutations were significantly older, predominantly male and more likely to have implantable cardioverter-defibrillators (ICDs) compared to those with *MYH7* mutations [28]. It suggests that the association between specific genotype and prognosis is affected by several factors such as type of variants, clinical risk factors and racial difference.

### Findings from Korean studies

Gwak et al. [29] investigated prognosis in 492 Korean patients with suspected HCM who underwent genetic testing. Disease-causing sarcomere mutations were detected in 43.5% of patients, with 40.7% classified as genotype-positive HCM. Genotype-positive patients experienced higher rates of composite adverse outcomes, including death, resuscitated arrest, heart failure admission, appropriate ICD shocks, and stroke (28.0% vs. 13.2%, *P* < 0.001), and sarcomere gene positivity remained an independent predictor of poor prognosis (hazard ratio, 1.70; 95% confidence interval [CI], 1.04–2.78; *P* = 0.034) in multivariable analysis. Kim et al. [30] evaluated the genotype–phenotype relationship and its prognostic implications in 89 Korean HCM patients, finding pathogenic or likely pathogenic variants in sarcomere genes (*MYBPC3, TNNI3, MYH7, MYL7*) in 27 patients, and overall genetic variants in 55 of 89 subjects (61.8%). Variant-positive patients exhibited higher rates of nonsustained ventricular tachycardia (30.0% vs. 12.5%; *P* = 0.030) and increased myocardial fibrosis on cardiac magnetic resonance (*P* = 0.029). Event-free survival was significantly lower in variant-positive patients (log-rank *P* = 0.006), demonstrating that genetic status provides independent prognostic information beyond clinical and imaging parameters.

### Oligogenic mutation and rare variant contribution

Girolami et al. [31] analyzed 488 unrelated HCM index patients screened across 8 sarcomeric genes and identified 4 individuals (0.8%) harboring rare triple sarcomere mutations (e.g., *MYH7-R869H, MYBPC3-E258K, TNNI3-A86fs*). Triple mutation carriers exhibited an aggressive clinical course, with most progressing to end-stage HCM by the fourth decade, necessitating transplantation or advanced pacing, and presenting higher rates of ventricular arrhythmias requiring ICD intervention. This work underscores the prognostic significance of multiple sarcomeric defects and supports comprehensive genetic panels for risk stratification in HCM.

### Inheritance patterns and genotype–phenotype correlations

HCM exhibits predominantly autosomal dominant inheritance with incomplete penetrance and variable expressivity. However, recently some variants have been shown to be related to autosomal recessive (*ALPK3*) or X-lined (*FHL1*) inheritance [4]. Lastly, data from the Mass General Brigham Biobank indicate that many genotype-positive but phenotype-negative individuals may remain clinically unaffected over extended follow-up, emphasizing the complex interplay of genetic and environmental factors in HCM penetrance [32].

### Modifier genes, epigenetics, and environmental interactions

While sarcomeric gene mutations serve as the primary drivers of HCM, they do not fully explain the disease's phenotypic heterogeneity. Modifier genes are those that do not directly cause HCM but influence the severity, age of onset, and associated complications are increasingly recognized for their role in modulating clinical expression. For example, variants in angiotensin-converting enzyme, transforming growth factor β, and ion channel genes have been implicated in myocardial remodeling and arrhythmogenesis [33]. Epigenetic mechanisms such as DNA methylation, histone acetylation, and noncoding RNAs may regulate gene expression in cardiomyocytes without altering the underlying DNA sequence. These changes can be driven by aging, comorbidities, or inflammation, and may interact with inherited mutations to shape disease progression. Peñarroya et al. [34] presented a unique pair-matched model, based on three monozygotic twin pairs carrying the same founder pathogenic variant and different phenotypes. This study provides further evidence of the pivotal role of epigenetics in HCM for variable expressivity. Environmental factors, including hypertension, physical exertion, and metabolic stress, also contribute significantly to disease variability. In genotype-positive/phenotype-negative individuals, high-intensity athletic training or chronic pressure overload may precipitate hypertrophy or arrhythmic events [35]. In the Korean National Health Insurance Registry data, a significant association was found between body mass index (BMI) and the incidence of clinical HCM after multivariate adjustment. The hazard ratio per 1 kg/m^2^ increase in BMI was 1.063 (95% CI, 1.051–1.075). Metabolically unhealthy participants had a higher incidence of HCM than metabolically healthy participants, regardless of obesity status. The effect of BMI was more pronounced in several subgroups, including participants without hypertension, those under 65 years of age, and men. Therefore, the authors suggested that efforts to manage obesity and metabolic abnormalities could modify the clinical expression of HCM [36, 37]. These results were consistent with a previous European study [37]. These gene–environment interactions underscore the need for tailored surveillance strategies.

## Role of genetic testing

### Diagnostic utility

Genetic testing is recommended for individuals with a clinical diagnosis of HCM, first-degree relatives of affected individuals, and asymptomatic at-risk family members [1].

### Genetic counseling

Genetic counseling is crucial for interpreting test results, understanding inheritance risks, and guiding surveillance strategies. ​

### Variants of uncertain significance

A challenge in clinical genetics is the interpretation of VUS, which requires integration of family history, phenotype, and emerging functional data. ​

## Family screening recommendations

### Genetic testing and counseling

#### Offer genetic testing to patients with HCM

Genetic testing should be offered to individuals diagnosed with HCM to identify pathogenic or likely pathogenic variants. This facilitates cascade testing in at-risk family members. ​

#### Counseling before and after genetic testing

Individuals undergoing genetic testing should receive genetic counseling before and after testing to understand the implications of test results. ​

### Cascade genetic testing in families

#### First-degree relatives

If a pathogenic or likely pathogenic variant is identified in a proband, cascade genetic testing should be offered to first-degree relatives (parents, siblings, and children). ​

#### Variants of uncertain significance

In cases where a VUS is identified, serial reevaluation of the variant's clinical significance is recommended, as reclassification may impact family screening strategies.

### Clinical screening for relatives

#### Initial evaluation

First-degree relatives should undergo a comprehensive clinical evaluation, including a 12-lead electrocardiogram (ECG) and a two-dimensional transthoracic echocardiogram (TTE).

#### Ongoing surveillance

For asymptomatic children and adolescents who are genotype-positive but phenotype-negative, clinical screening (ECG and TTE) is recommended every 1 to 2 years. ​For asymptomatic adults who are genotype-positive but phenotype-negative, clinical screening is recommended every 3 to 5 years. ​If a relative tests negative for the familial pathogenic variant, additional clinical screening is not recommended (Table 1) [1].

### Special considerations

#### Early-onset disease in family

In families with early-onset HCM or a history of SCD, earlier and more frequent screening may be warranted [38].

#### Athletes

Individuals involved in competitive sports should undergo thorough evaluation, as HCM is a leading cause of SCD in young athletes, although there are some controversies [39, 40].

## Implications for management and therapy

### Risk stratification

Genetic data can inform risk assessment for SCD, particularly in the presence of malignant mutations or family history of SCD.​ Ho et al. [25], a US-based team, have demonstrated genotype-associated outcomes and guided early-stage clinical trials for gene-targeted therapies.

### Genotype-guided therapy

Although current treatment remains largely phenotype-based (e.g., β-blockers, ICD placement), emerging therapies aim to target specific molecular pathways. ​

Mavacamten or aficamten, a myosin ATPase inhibitor, has shown promise in reducing hypercontractility in sarcomeric HCM [41, 42]. In sarcomere geno-positive patients, mavacamten achieved the primary composite endpoint with an OR of 4.43 (95% CI, 1.56–12.58), compared to an OR of 2.52 (95% CI, 0.99–6.42) in sarcomere geno-negative patients in the EXPLORER-HCM study [43]. A stronger and more sustained response has been observed in patients with pathogenic or likely pathogenic sarcomere variants. Therefore, genetic testing may be a useful tool for predicting therapeutic response and guiding personalized management strategies, including decisions regarding septal reduction therapies [44].

In a groundbreaking effort, the Cleveland Clinic initiated the first human gene therapy trial for HCM in 2023, targeting *MYBPC3* with an adeno-associated virus vector (TN-201). Preclinical data support reversal of disease manifestations through restoration of sarcomeric protein expression [45].

Gene-editing technologies (e.g., CRISPR/Cas9) and RNA-based therapies are being explored for mutation correction or silencing [46, 47].

## Cost-effectiveness of genetic test

One of the key hurdles in performing genetic testing is cost-effectiveness. Although the cost of genetic testing has decreased due to advances in next-generation sequencing technologies, the overall cost—including interpretation—remains relatively high. Additionally, insurance coverage (whether governmental or private) varies significantly across countries. However, considering current guidelines on cascade screening and regular clinical follow-up, several studies have shown that genetic testing is cost-effective [48–50]. This is because genetic testing allows for the identification of genotype-negative individuals among family members. According to current guidelines, regular ECG and echocardiographic follow-up are not recommended for these genotype-negative family members. In contrast, without genetic information, all first-degree relatives—starting from childhood—would require regular clinical surveillance, leading to unnecessary healthcare costs for asymptomatic individuals. Moreover, genetic testing can help identify high-risk patients earlier, potentially reducing the cost and burden associated with managing advanced disease stages.

## Future directions

The field is moving toward precision medicine in HCM, integrating genomics, transcriptomics, and proteomics to personalize care. Longitudinal studies and registries are essential to validate genotype–phenotype associations and assess the long-term impact of genetic interventions.

## Supplementary Information

Supplementary Material 1.

## Data Availability

No datasets were generated or analysed during the current study.

## Abbreviations

ACMG: American College of Medical Genetics and Genomics
BMI: Body mass index
CI: Confidence interval
ECG: Electrocardiogram
GLS: Global longitudinal strain
HCM: Hypertrophic cardiomyopathy
ICD: Implantable cardioverter-defibrillators
LVH: Left ventricular hypertrophy
mtDNA: Mitochondrial DNA
OR: Odds ratio
SCD: Sudden cardiac death
tRNA: Transfer RNA
TTE: Transthoracic echocardiogram
VUS: Variants of uncertain significance
